# Does electronic invoicing lead to stronger tax compliance? Evidence from China

**DOI:** 10.1371/journal.pone.0331880

**Published:** 2026-04-20

**Authors:** Ruicui Tao, Jiakun Li

**Affiliations:** 1 School of Economics and Management, Shanghai Polytechnic University, Shanghai, China; 2 School of Public Finance and Administration, Shanghai Lixin University of Accounting and Finance, Shanghai, China; University of Naples Federico II: Universita degli Studi di Napoli Federico II, ITALY

## Abstract

This article investigates whether China’s fully digitalized electronic invoicing reform has improved tax compliance, using a staggered difference-in-differences approach. The results show that: (1) The e-invoicing reform has significantly improved firms’ effective tax rate by 0.91 percentage points on average. This finding remains robust after controlling for firm-level and regional confounders and across a range of robustness checks, including alternative measures of tax compliance and alternative clustering of standard errors. (2) Mechanism analyses indicate that the reform reshapes firms’ reporting behavior on both the revenue and cost sides of the production process. Firms adjust reported revenues and reported costs jointly, with a stronger contraction in reported costs than in reported revenues, leading to an overall reduction in tax evasion. (3) Heterogeneity analyses reveal that the compliance-enhancing effect of the reform is more pronounced among non-state-owned enterprises, firms operating outside key industries under intense tax authority scrutiny, and those located in regions with lower tax enforcement capacity. This study provides micro-level evidence on the effectiveness of digital tax reforms and offers practical implications for tax governance policy.

## Introduction

Tax administration plays a central role in sustaining effective governance and long-term economic development. Beyond revenue collection, a transparent and credible tax system underpins social equity, limits rent-seeking behavior, and strengthens state capacity. Yet, as production networks become increasingly fragmented and business transactions more complex, tax authorities worldwide face growing difficulties in detecting corporate tax non-compliance. Traditional enforcement mechanisms—largely reliant on ex post audits and self-reported information—often suffer from delayed oversight, high administrative costs, and limited cross-verification capacity, reducing their effectiveness in curbing income misreporting, cost inflation, and fictitious invoicing.

In response to these challenges, governments across diverse institutional settings have increasingly turned to digital technologies to modernize tax administration. A common feature of these reforms is the expansion of third-party reporting, real-time data transmission, and automated cross-checking, which fundamentally reshape the information environment underlying tax enforcement. Despite their growing policy relevance, however, empirical evidence on the effectiveness of comprehensive digital tax enforcement systems remains limited. Existing studies typically examine partial targeted interventions, leaving open the question of how fully integrated digital regimes affect firms’ compliance behavior in practice.

Addressing this gap requires a setting in which digital tax enforcement is implemented in a comprehensive and coordinated manner, while still generating credible quasi-experimental variation. China provides such a setting. Rather than treating China as an exceptional case, we view it as a large-scale policy laboratory in which a fully digitalized e-invoicing system was rolled out in a staggered fashion across regions. This institutional feature allows us to study how an integrated digital invoicing regime affects firms’ reporting behavior under real-world constraints.

The fully digitalized e-invoicing reform mandates that all invoices be issued, transmitted, and archived through a unified national platform using structured data formats. Its core design features automatic archiving upon issuance and instant verification upon archiving. By construction, this architecture limits managerial discretion over invoice timing and content, reduces opportunities for fictitious or delayed invoicing, and enhances transaction traceability. Moreover, real-time access to invoice-level data strengthens tax authorities’ informational advantage, enabling anomaly detection, cross-verification across trading partners, and more targeted audits, thereby increasing the expected cost of tax evasion. The reform was introduced through pilot programs in 2021 and subsequently expanded across regions before being institutionalized nationwide. We exploit this staggered rollout and apply a multi-period difference-in-differences design to identify the causal effect of the reform on corporate tax compliance, using firm-level panel data on Chinese A-share listed companies from 2019 to 2023.

Our analysis addresses three questions. First, does a fully digitalized e-invoicing system improve firms’ tax compliance, as measured by effective tax rates? Second, how does digital tax enforcement reshape firms’ reporting behavior along different margins of the production process, including revenue reporting and cost deductions? Third, do compliance responses vary systematically across firms with different ownership structures, industry characteristics, and local enforcement capacities?

We find that the digital e-invoicing reform significantly improves corporate tax compliance, increasing firms’ effective tax rates by approximately 0.91 percentage points. Mechanism analyses show that this effect operates through joint adjustments in firms’ reported revenues and reported costs. Following the reform, reported revenues decline by about 1.90%, while reported costs decrease by 2.08%, indicating that firms realign their reporting of outputs and inputs in response to tighter digital enforcement. Importantly, the contraction in overstated costs dominates the reduction in reported revenues, leading to an overall expansion of the taxable base and a reduction in tax evasion. The effects are more pronounced among non-state-owned enterprises, firms in less heavily regulated industries, and regions with lower enforcement capacity, highlighting substantial heterogeneity in behavioral responses.

This paper contributes to the literature on tax compliance and digital tax enforcement by providing causal evidence on how a fully digitalized, information-integrated e-invoicing system reshapes firms’ reporting behavior and compliance outcomes. Exploiting the staggered regional rollout of China’s e-invoicing reform and rich firm-level panel data, we identify the impact of a comprehensive change in the enforcement information structure, rather than isolated digital tools, on effective tax rates. We further unpack the underlying mechanisms by showing that the compliance effect operates primarily through enhanced income transparency and tighter verification of deductible costs, with little impact on firms’ underlying profitability, and that responses vary systematically across ownership types, industry regulation, and local enforcement capacity. Although the empirical setting is China, the mechanisms we study are general to information-based tax enforcement and therefore offer broader insights for economies expanding real-time reporting, third-party information integration, and data-driven tax administration.

## Literature review

This paper contributes to three strands of the literature on tax compliance and digital tax enforcement. Specifically, it builds on studies examining (i) firms’ behavioral responses to digitalized tax enforcement, (ii) the role of institutional and informational constraints in shaping evasion capacity, and (iii) the governance conditions and operational limits under which tax technologies operate.

### Behavioral responses to digitalized tax enforcement

This stream of literature focuses on how firms strategically respond to enhanced digital oversight mechanisms, such as real-time invoice monitoring and structured electronic reporting. Rather than emphasizing the technology itself, these studies examine changes in firms’ reporting behavior, profit shifting, and compliance strategies.

Bellon et al. (2022) [1] find that the adoption of e-invoicing in Peru increases reported sales, purchases, and VAT liabilities by over 5% in the first year, especially among small firms and low-compliance sectors. However, many firms simultaneously use accumulated VAT credits to offset tax payments, limiting the net revenue effect. Kotsogiannis et al. (2025) [2] show that e-invoicing improved net VAT payments in Rwanda mainly by enhancing audit capabilities, not voluntary compliance. In the Chinese context, Zhang et al. (2020) [3] find that the Golden Tax Phase III system significantly reduced tax evasion by improving invoice traceability. Li et al. (2020) [4] further demonstrate that the tax credit rating system within this institutional setting influences both compliance and firm-level financial strategies, suggesting broad behavioral consequences of digital enforcement. Zhang and Fang (2024) [5] document that large business groups shift profits from digitally supervised pilot regions to non-pilot regions under China’s digital tax supervision regime to avoid tax scrutiny, highlighting strategic intra-group adjustments under policy pressure.

### Institutional and informational constraints on evasion capacity

This group of studies examines how tax compliance is shaped by institutional design and the structure of information flows. A central theme is the deterrent power of third-party verification and system-based audit credibility.

The foundational model by Allingham and Sandmo (1972) [6] conceptualizes tax evasion as an optimal choice under risk, shaped by audit probabilities, penalty severity, and risk aversion. Kleven et al. (2011) [7] provide empirical validation that third-party reporting in Denmark sharply reduces underreporting. Pomeranz (2015) [8] shows that value-added tax systems in the Chilean setting exhibit self-enforcing characteristics when third-party information is embedded in invoice chains. Desai and Dharmapala (2006,2009) [9,10] highlight that corporate tax avoidance is moderated by agency conflicts and governance quality. In the Chinese context, Tian et al. (2021) [11] find that invoice lottery incentives significantly strengthen third-party mechanisms in service sectors. Zhao et al. (2025) [12] show that digital tools, such as online invoice verification with consumer rewards, within this institutional environment, further enhance real-time information flow and improve enforcement outcomes.

### Governance conditions and operational limits of tax technology

This stream focuses on the institutional prerequisites and real-world limitations of technology-based tax enforcement. While digital tools are often celebrated for their potential, these studies caution that their effectiveness depends critically on implementation capacity, taxpayer literacy, and governance context.

Mascagni et al. (2023) [13] find inconsistencies between electronic billing machine records and tax declarations in Rwanda, mainly due to user error and operational constraints. Casey and Castro (2015) [14] argue that electronic fiscal devices are not a silver bullet and require complementary governance reforms and user training. Using cross-country data, Kochanova et al. (2020) [15] document that e-filing reduces time spent on tax tasks and improves perceptions of tax authorities, though its impact on revenue is modest. Okunogbe and Pouliquen (2022) [16] show that, using firm-level data from Tajikistan, replacing in-person filing with e-filing reduced compliance costs, tax liabilities, and the incidence of bribery. Dzansi et al. (2022) [17] show that mobile and geospatial technologies helped local tax authorities in Ghana increase tax collection efficiency. Mascagni et al. (2021) [18] find that real-time sales registration improved VAT–income tax consistency and audit quality in Ethiopia.

Building on the above research, these studies highlight both the opportunities and the limitations of digital tax tools. While third-party information and automation can improve compliance, their success is contingent on institutional quality, technological readiness, and taxpayer responsiveness. Despite the growing global literature, few studies have rigorously evaluated national-level, fully digitalized invoicing systems like that of China. This paper contributes by providing micro-level causal evidence on the compliance effects of China’s comprehensive e-invoicing reform.

## Research design and data

### Research design and variable measurements

To empirically evaluate the impact of China’s digital electronic invoicing reform on tax compliance, this study exploits the staggered implementation of the policy across provinces and over time. Beginning in 2021, the State Taxation Administration rolled out the fully digitalized electronic invoicing reform on a pilot basis in selected regions, gradually expanding it nationwide. This variation in policy timing and geographic coverage provides a quasi-natural experimental setting suitable for causal inference. Our analysis focuses on firms’ compliance responses induced by the adoption of e-invoicing in their own registered regions, capturing changes in firm-level reporting constraints rather than modeling the completeness of invoice-chain enforcement across transaction partners during the staggered rollout.

Accordingly, the baseline estimation adopts a Difference-in-Differences (DID) framework, specified as follows:


$$y_{it}=\beta_{0}+\beta_{1}digital_{ijt}+\beta_{2}control_{ijt}+\varphi_{i}+\eta_{t}+\varepsilon_{ijt}$$ (1)

In the above specification, *y*_*it*_ measures firm-level tax compliance, *digital*_*ijt*_ indicates whether firm i in region j is exposed to the digital e-invoicing reform in year t and *control*_*ijt*_ accounts for both firm-level financial characteristics and regional macroeconomic conditions. To address unobserved heterogeneity, the model includes firm fixed effects ($\varphi_{i}$) to control for time-invariant characteristics such as ownership structure or management quality, and year fixed effects ($\eta_{t}$) to capture nationwide shocks or macroeconomic trends. The coefficient of interest $\beta_{1}$ estimates the effect of the e-invoicing policy on corporate tax compliance. A positive and statistically significant $\beta_{1}$ would suggest that the reform contributes to higher tax compliance among affected firms.

The dependent variable is tax compliance, proxied by the Effective Tax Rate (ETR). As a systemic digital transformation of invoice administration, China’s digital electronic invoicing reform affects both the issuance and receipt of invoices, thereby influencing firms’ overall tax-related behavior in a comprehensive manner. To capture this effect, we follow standard practices in the literature [19–21] and define ETR as the ratio of a firm’s income tax expense to its pre-tax earnings. A higher ETR typically indicates a smaller gap between the actual and statutory tax burden, signaling a higher degree of tax compliance. As a result, ETR is widely regarded as a core metric for evaluating firms’ adherence to income tax obligations.

Importantly, prior studies suggest that when tax reserves and deferred tax assets are not explicitly accounted for, ETR serves as the most informative proxy—on average—for identifying firms’ uncertain tax avoidance behavior [22]. In this context, adopting ETR as the outcome variable enables us not only to assess how the reform affects compliance behavior but also to indirectly detect shifts in aggressive tax planning practices.

The key explanatory variable is the policy treatment dummy (digital), which equals 1 if firm i, located in region j, is exposed to the e-invoicing reform in year t; otherwise, it is set to 0. This variable captures whether a firm is subject to the reform and is central to the staggered DID identification strategy used in this study.

In selecting control variables, we follow prior studies including Gupta and Newberry (1997) [19], Liu and Liu (2014) [23], Liu and Ye (2018) [24], Zhang et al. (2024) [5], and Zhao et al. (2025) [12]. To mitigate potential confounding factors, we control for both firm-level and region-level characteristics that may jointly influence firms’ tax behavior and policy exposure. At the firm level, we include variables such as firm size, leverage, cash holdings, asset depreciation, capital intensity, and intangible assets ratio—factors known to shape tax planning incentives and financial flexibility. At the regional level, we account for broader economic and institutional conditions by incorporating the logarithm of provincial GDP (as a proxy for regional development), the local fiscal self-sufficiency ratio (measured as the ratio of general public revenue to expenditure), and the regional industrial structure (captured by the ratio of tertiary to secondary sector output). These controls are intended to absorb regional variation in enforcement intensity and economic environments, thereby enhancing the credibility of our causal estimates.

The full definitions of all variables used in the analysis are provided in S1 Table.

### Data description

This study examines all non-financial A-share listed firms in China over the period 2019–2023. Firm-level financial and governance data are drawn from the China Stock Market & Accounting Research (CSMAR) database, while provincial-level economic and fiscal indicators are obtained from its macroeconomic module. Focusing on A-share listed firms allows us to leverage high-quality, standardized financial information subject to relatively stringent disclosure and enforcement requirements, thereby enhancing the internal validity of the empirical analysis. As with most studies based on listed firms, the extent to which the findings generalize to smaller or unlisted firms should be interpreted with appropriate caution.

Following established practices in the literature (e.g., Zhang et al., 2020; Chen et al., 2023; Zhao et al., 2025) [3,12,25], we conduct several data cleaning steps to ensure the reliability of the empirical analysis. Specifically, we exclude firms flagged as ST or *ST, which are designations applied by Chinese stock exchanges to firms experiencing severe financial distress or facing delisting risk. Observations with negative depreciation of fixed assets or effective tax rates exceeding 1 are removed (Chen et al., 2010; DeFond et al., 2025; Zhang and Yang, 2025) [26–28]. We also drop firms with “actual tax burdens” — calculated as income tax payable divided by operating revenue — that are less than 0 or greater than 1 (Zhang et al., 2020) [3], as such values suggest data anomalies. In addition, firms with negative pre-tax income are excluded (Zhao et al., 2025; Fan et al., 2025) [12,29], as tax compliance indicators are not meaningful in such cases.

To further mitigate the influence of outliers, all continuous variables are winsorized at the 1st and 99th percentiles. This ensures the robustness of the regression results by reducing the distortion caused by extreme values. After applying the data cleaning procedures and variable construction, the final estimation sample comprises 15,652 firm-year observations from 2019 to 2023.

Table 1 presents descriptive statistics for the key variables. The mean effective tax rate is 16.1%, which is notably below the statutory rate of 25%, suggesting that firms either benefit from preferential tax policies or engage in tax avoidance. On average, 27.8% of the firms in the sample are exposed to the e-invoicing reform in a given year, indicating substantial coverage of the policy shock. The average financial leverage ratio is 38.6%, capital intensity stands at 19.0%, and intangible assets ratio is approximately 4.5%.

**Table 1:** Descriptive statistics of main variables.

Variable Name	N	Mean Value	Standard Deviation	Minimum	Maximum
etr	15640	0.161	0.106	0.000	0.636
digital	15652	0.278	0.448	0.000	1.000
size	15652	22.366	1.344	19.973	26.401
lev	15652	0.386	0.189	0.056	0.836
cash	15652	0.180	0.132	0.014	0.635
lnfad	15652	18.014	1.658	14.279	22.545
capital	15652	0.190	0.144	0.002	0.654
intang	15581	0.045	0.064	0.000	0.936
ziji	15652	0.620	0.171	0.211	0.922
chanye	15652	1.758	1.138	0.900	5.690
lngdp	15652	29.375	0.657	26.952	30.239

### Empirical analysis

Table 2 reports the baseline difference-in-differences estimates in Columns (1)–(2), followed by a set of robustness checks in Columns (3)–(5). Across specifications, the coefficient on the digital e-invoicing reform is statistically significant, indicating an improvement in firms’ tax compliance.

**Table 2:** Baseline and robustness results.

	Baseline results		Robustness tests		
Variable	(1) ETR	(2) ETR	(3) ETR2	(4) Taxdiff	(5) ETR
digital	0.00926***	0.00910***	0.00104*	−0.00898***	0.00910***
	(0.00325)	(0.00307)	(0.000539)	(0.00303)	(0.00336)
size		−0.00900**	0.00564***	0.0127***	−0.00900*
		(0.00353)	(0.00101)	(0.00402)	(0.00466)
lev		0.0453**	−0.0241***	−0.0508**	0.0453***
		(0.0210)	(0.00555)	(0.0211)	(0.0130)
cash		−0.00655	0.0102***	0.00792	−0.00655
		(0.0154)	(0.00291)	(0.0149)	(0.00889)
lnfad		0.00769**	−0.00207**	−0.00808***	0.00769***
		(0.00312)	(0.000769)	(0.00278)	(0.00274)
capital		0.0118	−0.0173***	−0.00345	0.0118
		(0.0204)	(0.00439)	(0.0220)	(0.0168)
intang		0.0595	−0.0249*	−0.0630	0.0595*
		(0.0374)	(0.0137)	(0.0415)	(0.0316)
ziji		0.0122	0.0230***	−0.0187	0.0122
		(0.0595)	(0.00715)	(0.0593)	(0.0391)
chanye		−0.000530	−0.00141	−0.0000770	−0.000530
		(0.00495)	(0.00111)	(0.00523)	(0.00529)
lngdp		0.0157	0.00163	−0.0226	0.0157
		(0.0227)	(0.00535)	(0.0233)	(0.0258)
_cons	0.159***	−0.268	−0.116	0.582	−0.268
	(0.000876)	(0.638)	(0.153)	(0.648)	(0.754)
Firm FE	Yes	Yes	Yes	Yes	Yes
Year FE	Yes	Yes	Yes	Yes	Yes
Observations	14,928	14,860	14,860	14,474	14,860
Adj. *R*^2^	0.5530	0.5563	0.6420	0.4595	0.5563

Notes: * p < 0.1, ** p < 0.05, *** p < 0.01. Standard errors in parentheses.Columns (1), (2), and (5) use ETR as the dependent variable; Column (3) uses ETR2 (income tax expense divided by operating revenue); Column (4) uses Taxdiff (statutory minus effective tax rate). Standard errors are clustered at the province level in Column (2) and at the firm level in Column (5).

Column (1) presents the baseline specification with firm and year fixed effects, capturing within-firm changes in tax compliance around the adoption of the digital e-invoicing reform. Column (2) augments this specification by further controlling for a comprehensive set of firm-level financial characteristics and regional macroeconomic conditions. The estimated coefficient on the reform indicator remains positive and statistically significant, with a comparable magnitude across the two specifications. In the preferred specification reported in Column (2), the adoption of digital e-invoicing is associated with an increase of approximately 0.91 percentage points in the effective tax rate relative to the pre-reform mean, indicating an economically meaningful improvement in firms’ tax compliance.

To ensure the robustness and reliability of our findings, this study conducts three additional tests beyond the baseline regression analysis. First, we address concerns regarding the measurement of the dependent variable. While the effective tax rate is a widely accepted indicator of tax compliance, it is calculated based on pre-tax income, which may be affected by fluctuations in profitability or accounting adjustments. To mitigate potential bias arising from this, we adopt an alternative measure—following the approach of Fan and Peng(2017) [30] —by using the ratio of income tax expense to operating revenue (hereafter, ETR2). This alternative proxy reduces sensitivity to profit volatility and more directly reflects a firm’s tax burden relative to its sales, offering a complementary perspective on tax compliance behavior.

As shown in Column (3) of Table 2, using ETR2 as an alternative measure yields a positive and statistically significant effect of the reform. Although ETR2 normalizes income tax payments by operating revenue rather than pre-tax income, the estimated effect is of comparable magnitude to that in the baseline specification, indicating that the main results are robust to alternative measures of tax compliance.

Second, to further account for heterogeneity in allowable deductions and firm-specific tax planning opportunities, we construct an alternative dependent variable defined as the difference between the statutory corporate income tax rate and the effective tax rate (Chen et al., 2010; Xu and Li, 2020; Zhou and Cao, 2025; Chen and Liu, 2025) [26,31–33]. This measure captures the extent to which firms’ actual tax burdens deviate from statutory benchmarks.

As shown in column (4) of Table 2, the coefficient on the reform variable remains negative and statistically significant, indicating that firms’ tax burdens move closer to statutory tax liabilities after the implementation of e-invoicing. This result confirms that our main findings are not driven by the specific construction of ETR.

Third, we address the potential concern that clustering standard errors at the provincial level—though justified given the reform’s province-level implementation—might not adequately account for intra-firm correlations in tax behavior. Factors such as internal control mechanisms, financial strategies, or managerial preferences could introduce firm-level heterogeneity that is not captured at the provincial level. To address this, we re-estimate the model with standard errors clustered at the firm level.

Column (5) of Table 2 reports the results of this alternative specification. The coefficient on the treatment variable remains positive and statistically significant at the 1% level. Since changing the clustering level affects only the standard errors and not the point estimates, this result indicates that the statistical significance of the estimated policy effect is robust to alternative clustering choices.

Taken together, the results based on alternative outcome measures and clustering strategies consistently support our main conclusion: the digital e-invoicing reform leads to a statistically and economically meaningful improvement in firms’ tax compliance. These results enhance the empirical credibility of our study and provide compelling evidence of the reform’s effectiveness in advancing modern tax administration in China.

Finally, we test the common trend assumption underlying the DID design. The validity of the DID approach requires that, absent the reform, treated and control firms would have followed similar trends in tax compliance. In our setting, this implies that treated and untreated firms should display comparable pre-policy trends in the ETR.

**Fig 1:**
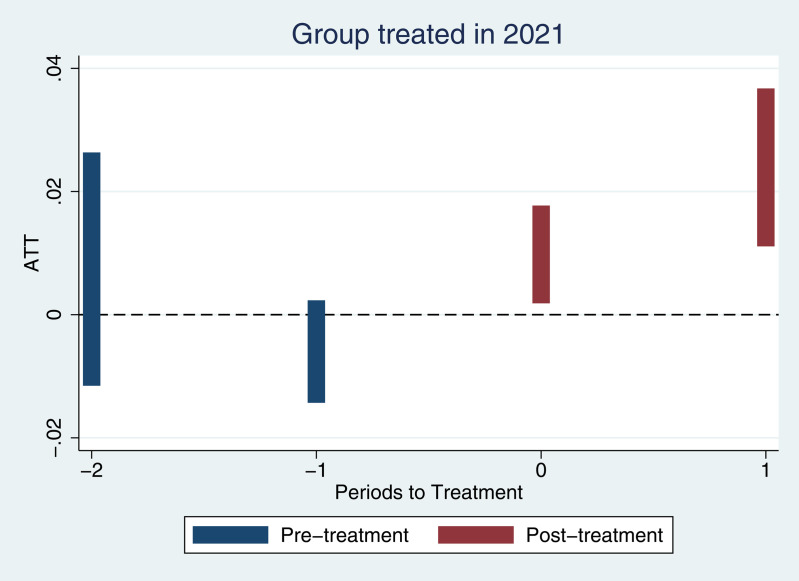
Event study estimates for parallel trends validation. This figure plots the event study estimates of average treatment effects (ATT) around the adoption year (2021). Blue bars represent pre-treatment periods (*t* = −2,−1), while red bars represent post-treatment periods (*t* = 0,1). The vertical lines denote 95% confidence intervals.

To evaluate this assumption, we apply an event-study framework following Callaway and Sant’Anna (2021) [34], which allows us to examine dynamic treatment effects relative to the timing of policy adoption. As shown in Fig 1, the average treatment effects on the treated (ATT) in the pre-treatment periods (t = –2 and t = –1) are statistically indistinguishable from zero, with the confidence intervals encompassing the null value. This indicates that no significant differences in tax compliance trends existed between treated and untreated firms before the policy intervention, thereby supporting the validity of the parallel trends assumption.

Following the introduction of the policy, however, we observe statistically significant and positive ATT estimates in both the implementation year (t = 0) and the following year (t = 1). This post-treatment increase in ETR suggests that the digital e-invoicing reform effectively enhanced firms’ tax compliance behavior. The observed pattern—stable outcomes before treatment and a sharp change thereafter—is consistent with the identifying assumptions of the DID strategy and provides a solid foundation for causal inference.

### Mechanism of the impact of the fully digitalized e-invoicing policy on tax compliance

To shed light on the mechanisms through which the fully digitalized e-invoicing reform improves corporate tax compliance, this section examines how the policy reshapes firms’ financial reporting behavior on both the revenue and cost sides of the production process. The mechanism analysis focuses on changes in firm-level reporting constraints induced by the adoption of e-invoicing in firms’ own registered regions. By altering firms’ reporting environment, the reform is expected to constrain accounting discretion and induce adjustments in both reported revenues and deductible costs.

We focus on three outcome variables as proxies for firms’ reporting behavior and performance: the natural logarithm of reported operating revenue (lnrevenue), the natural logarithm of reported operating costs (lncost), and return on assets (roa). Changes in lnrevenue and lncost capture adjustments in firms’ reporting on the output and input sides, respectively, while roa reflects whether the reform affects firms’ underlying profitability and operational efficiency.

Table 3 reports the corresponding regression results. In Column (1), we observe that the coefficient on the treatment variable is equal to −0.0190 and statistically significant at the 5% level. This implies that, following the reform, treated firms experienced an average decrease of 1.90% in reported revenue. Importantly, this effect holds after controlling for firm size, capital structure, leverage and regional characteristics. Taken in isolation, a reduction in reported revenues could be interpreted as increased underreporting. However, this interpretation changes once revenues and costs are jointly considered. As shown in column (2), the estimated coefficient on the treatment variable is negative and statistically significant at the 5% level. This result implies that, following the reform, treated firms report on average 2.08% lower operating costs than their pre-reform baseline. We interpret this as evidence that the reform curtails the use of inflated or fictitious invoices to overstate deductible expenses. This pattern suggests that firms adjusted both the input and output sides of their production process, realigning reported revenues and costs toward more accurate levels. Consistent with this interpretation, ETR increases after the reform, implying that the contraction in overstated costs dominates the reduction in reported revenues, leading to an overall decrease in tax evasion.

**Table 3:** Mechanism analysis.

Variable	(1) lnrevenue	(2) lncost	(3) roa
digital	−0.0190**	−0.0208**	−0.000344
	(0.00863)	(0.00786)	(0.00140)
size	0.710***	0.686***	−0.00680**
	(0.0221)	(0.0258)	(0.00279)
lev	0.295**	0.409***	−0.0423***
	(0.111)	(0.107)	(0.0124)
cash	−0.0454	−0.148***	0.0147***
	(0.0406)	(0.0415)	(0.00425)
lnfad	0.0826***	0.103***	−0.000103
	(0.0175)	(0.0209)	(0.00142)
capital	−0.0732	−0.000117	−0.0515***
	(0.0766)	(0.106)	(0.00772)
intang	−0.250**	−0.208	−0.0579**
	(0.107)	(0.135)	(0.0268)
ziji	0.290***	0.162	0.0491**
	(0.100)	(0.0970)	(0.0219)
chanye	0.0220	0.0255**	−0.00155
	(0.0156)	(0.0121)	(0.00274)
lngdp	0.0806	0.0871	0.00453
	(0.0962)	(0.126)	(0.0125)
Constant	1.701	1.276	0.0788
	(2.851)	(3.629)	(0.347)
Firm FE	Yes	Yes	Yes
Year FE	Yes	Yes	Yes
Observations	14,874	14,874	14,874
Adj. *R*^2^	0.9817	0.9815	0.6097

*Note:* * *p* < 0.1, ** *p* < 0.05, *** *p* < 0.01. Standard errors are reported in parentheses.

In Column (3), the regression with roa as the dependent variable yields a treatment effect of −0.000344, which is statistically insignificant. This suggests that, despite the decline in reported revenue, the reform did not have a meaningful impact on firms’ actual profitability or efficiency in utilizing their assets. This finding is important because it indicates that the reform primarily affects financial reporting behavior rather than real economic performance.

Furthermore, this finding aligns with existing theoretical models that emphasize the role of enforcement technology in shaping firm behavior. Enhanced visibility into transactions reduces information asymmetry between firms and regulators, thereby lowering the returns to evasion and increasing the expected cost of misreporting. The results also resonate with prior empirical work suggesting that digital tax administration tools are more effective in curbing aggressive accounting practices than in altering core business operations (e.g., Naritomi, 2019; Pomeranz,2015) [8,35].

### Heterogeneous effects

All heterogeneous effects reported in this section are estimated using the baseline specification, with heterogeneity examined through sample splits or subgroup analyses.

To further investigate the heterogeneous effects of the digital e-invoicing reform, we first examine whether the policy effect varies by ownership type. Specifically, we divide the sample into state-owned enterprises (SOEs) and non-state-owned enterprises (non-SOEs). Prior research suggests that non-SOEs typically exhibit stronger incentives for tax avoidance and lower baseline levels of tax compliance compared to their state-owned counterparts (Cai & Liu, 2009) [36]. Consequently, they may be more responsive to enhanced oversight mechanisms, particularly those enabled by digital technologies. The full-process digitalization of invoicing imposes stricter constraints on traditional evasion strategies—such as inflating deductible costs or underreporting revenue—by increasing data transparency and improving real-time traceability. Therefore, it is expected that the reform would exert a more substantial compliance effect on non-SOEs.

Columns (1)–(2) of Table 4 show that the the estimated coefficient on the reform indicator is positive and statistically significant at the 5% level for non-SOEs, but statistically insignificant for SOEs, suggesting that the overall effect is driven by non-SOEs. The magnitude for non-SOEs is comparable to that in the baseline specification.

**Table 4:** Heterogeneous effects.

	Ownership types		Industries		Tax enforcement intensity	
Variable	(1) Non-SOE	(2) SOE	(3) Key-regulated	(4) Non-key-regulated	(5) High TE	(6) Low TE
digital	0.00776**	0.0126	0.00686	0.0108**	0.00632**	0.00983*
	(0.00299)	(0.00857)	(0.00662)	(0.00418)	(0.00228)	(0.00505)
size	−0.0120***	−0.00703	−0.0288**	−0.00273	−0.00744	−0.0129
	(0.00428)	(0.0109)	(0.0107)	(0.00332)	(0.00681)	(0.00965)
lev	0.0298	0.0851***	0.0564	0.0421*	0.0276	0.0637**
	(0.0223)	(0.0295)	(0.0469)	(0.0232)	(0.0355)	(0.0263)
cash	0.00577	−0.0820**	−0.0348	0.00441	0.00726	−0.0102
	(0.0160)	(0.0339)	(0.0305)	(0.0153)	(0.0245)	(0.0161)
lnfad	0.00630	0.0128***	0.0117	0.00535**	0.00987**	0.00537
	(0.00395)	(0.00453)	(0.00821)	(0.00196)	(0.00417)	(0.00456)
capital	0.0198	−0.00942	−0.0237	0.0325	0.0263	−0.0101
	(0.0209)	(0.0499)	(0.0442)	(0.0233)	(0.0363)	(0.0274)
intang	0.0598	0.0157	−0.0220	0.0736*	0.106	0.0248
	(0.0519)	(0.0565)	(0.0544)	(0.0419)	(0.0636)	(0.0498)
ziji	0.0910	−0.0988	0.0791	−0.00343	0.0227	−0.0510
	(0.0678)	(0.101)	(0.0754)	(0.0656)	(0.0778)	(0.0489)
chanye	0.00632	−0.00799	0.00617	−0.00124	−0.00154	0.0202
	(0.00599)	(0.00797)	(0.0183)	(0.00401)	(0.00397)	(0.0311)
lngdp	0.0278	−0.0154	−0.0508	0.0484	−0.0527	−0.0199
	(0.0294)	(0.0608)	(0.0400)	(0.0349)	(0.0394)	(0.0458)
Constant	−0.602	0.603	2.054*	−1.335	1.666	0.913
	(0.833)	(1.727)	(1.159)	(1.000)	(1.174)	(1.376)
Firm FE	Yes	Yes	Yes	Yes	Yes	Yes
Year FE	Yes	Yes	Yes	Yes	Yes	Yes
Observations	10,483	4,292	3,536	11,233	6,237	5,891
Adj. *R*^2^	0.4930	0.6228	0.5718	0.5238	0.5963	0.5348

*Note:* * *p* < 0.1, ** *p* < 0.05, *** *p* < 0.01. Standard errors are reported in parentheses.

We next explore whether the effect of the digital invoicing reform varies across industries with different levels of regulatory scrutiny by dividing the sample into key-regulated industries (e.g., real estate, construction, medicine) and non-key-regulated industries based on classifications from the China Securities Regulatory Commission (2012 edition) and relevant tax policy documents.

Columns (3)–(4) of Table 4 show that the estimated coefficient is positive but not statistically significant in key-regulated industries, whereas it is positive and statistically significant at the 5% level in non-key-regulated industries. This suggests that the overall effect is driven by firms in non-key-regulated industries.

Finally, to investigate whether the policy effect varies across different institutional environments, we construct a tax enforcement intensity index (TE) based on the residual between actual and fitted tax burden ratios, following Lotz and Morss (1967) [37], Chelliah et al.(1975) [38], Mertens (2003) [39], Zeng and Zhang (2009) [40], Ye and Liu (2011) [41], and Jiang (2013) [42]. Specifically, we estimate the benchmark tax burden ratio using a cross-sectional model:


$$\frac{T_{jt}}{Y_{jt}}=\alpha +\beta_{1}GDP_{jt}+\beta_{2}IND1_{jt}+\beta_{3}IND2_{jt}+\varepsilon_{jt}$$ (2)

where *T*_*jt*_ denotes the tax revenue of region j in year t, *Y*_*jt*_ is its GDP, *GDP*_*jt*_ is the log of per capita GDP, and *IND*1_*jt*_ and *IND*2_*jt*_ represent the shares of primary and secondary industry in GDP, respectively. The fitted value represents the expected tax burden given the region’s economic fundamentals. The ration between the actual and fitted tax burden ratio is used as a proxy for local tax enforcement intensity.

We then classify cities into high and low enforcement groups based on whether their TE index exceeds the annual median. A dummy variable TE_DUM is assigned the value of 1 for cities above the median (high enforcement) and 0 otherwise (low enforcement).

Columns (5)–(6) of Table 4 show that, in regions with relatively high enforcement, the estimated coefficient on the reform indicator is positive and statistically significant. The coefficient remains positive and statistically significant at the 10% level in low-enforcement regions, with a larger magnitude. This suggests that the overall effect is stronger in regions with weaker baseline tax enforcement.

## Conclusions and policy implications

### Conclusions

This study exploits the staggered rollout of China’s fully digitalized electronic invoicing reform to examine its effect on corporate tax compliance within a multi-period difference-in-differences framework. The results indicate that the reform significantly improves tax compliance, as reflected in a higher effective tax rate after adoption. This finding remains robust to a range of alternative specifications and supplementary tests.

The mechanism evidence suggests that the compliance effect operates primarily through changes in firms’ reporting behavior rather than through changes in real operating performance. Following the reform, both reported revenues and reported costs decline, but the reduction in reported costs is larger, while profitability remains broadly unchanged. This pattern is consistent with the interpretation that digitalized invoicing strengthens transaction traceability and verification, limits invoice-based manipulation and cost overstatement, and thereby expands the taxable base.

The compliance effect varies across firms, industries, and regions, with stronger effects among non-state-owned enterprises, in less regulated industries, and in regions with weaker pre-existing tax enforcement. This pattern suggests that digital tax administration is particularly effective where traditional monitoring was previously less intensive.

### Policy implications

These findings have direct implications for China’s ongoing tax modernization agenda and for other developing economies seeking to strengthen revenue administration through digital governance. As fully digitalized electronic invoicing has already been rolled out nationwide in China, evaluating its policy consequences is particularly relevant. The evidence in this study suggests that digital invoicing can enhance tax compliance by reducing information frictions, improving transaction-level verification, and strengthening administrative oversight.

At the same time, the results indicate that digital tools are most effective when embedded in a broader institutional framework. The larger effects observed in weaker-enforcement environments imply that digital reform can partially compensate for limitations in traditional monitoring capacity, but cannot fully substitute for administrative capability. To consolidate the long-run benefits of the reform, tax authorities should further improve data integration, invoice cross-checking, inter-agency information sharing, and risk-based enforcement.

In addition, policymakers should pay attention to implementation capacity and compliance costs, especially for smaller firms facing greater technical adjustment burdens. Strengthening taxpayer services, system compatibility, and digital support mechanisms will be important for sustaining reform effectiveness. Overall, the study highlights that the value of digital tax reform lies not only in technological adoption itself, but also in its ability to support a more transparent, coordinated, and information-based system of tax governance.

Future research could extend this analysis to a broader range of firms and industry sectors, explore the long-term dynamics of tax compliance behavior, and investigate the interaction effects between digital technologies and other tax policy tools. Such efforts would contribute to a deeper and more comprehensive understanding of the modernization of tax governance.

## Supporting information

S1 Table Variable definitions. (DOCX)

S1 Appendix Additional methodological details. (DOCX)
