# Supplementary material for: Does electronic invoicing lead to stronger tax compliance? Evidence from China
Source: PLoS One. 2026 Apr 20;21(4):e0331880. doi: 10.1371/journal.pone.0331880 (PMC13095105; doi:10.1371/journal.pone.0331880)
Supplement: S1 Table — (DOCX) [file pone.0331880.s001.docx]

S1 Table. Variable definitions

| Variable Name | Definition |
| --- | --- |
| **etr** | Income tax expense divided by total profit (Effective Tax Rate) |
| **digital** | Dummy variable indicating implementation of digital e-invoicing reform |
| **size** | Natural logarithm of total assets at year-end |
| **lev** | Total liabilities divided by total assets at year-end (Leverage ratio) |
| **cash** | Cash and cash equivalents divided by total assets |
| **lnfad** | Natural logarithm of depreciation of fixed assets at year-end |
| **capital** | Net fixed assets divided by total assets at year-end |
| **intang** | Net intangible assets divided by total assets at year-end |
| **lngdp** | Natural logarithm of provincial GDP |
| **ziji** | Ratio of general public budget revenue to general public budget expenditure (Fiscal self-sufficiency) |
| **chanye** | Ratio of tertiary industry GDP to secondary industry GDP at the provincial level |
