# Supplementary material for: Does electronic invoicing lead to stronger tax compliance? Evidence from China
Source: PLoS One. 2026 Apr 20;21(4):e0331880. doi: 10.1371/journal.pone.0331880 (PMC13095105; doi:10.1371/journal.pone.0331880)
Supplement: S1 Appendix — (DOCX) [file pone.0331880.s002.docx]

**S1 Appendix.**

This appendix provides supplementary analyses that support the identification strategy and interpretation of the main results. Appendix A documents the sample construction and data cleaning procedures. Appendix B addresses potential confounding policies. Appendix C focuses on non-regulated industries, reporting summary statistics, baseline and robustness regressions, and event-study estimates.

**Appendix.A Sample Construction, Data Cleaning, and Distributional Diagnostics**

This appendix provides a transparent account of the sample selection process and documents how data screening affects the distributional properties of the key outcome variable. It reports (i) the precise observations excluded at each stage and the economic rationale for doing so, (ii) a comparison of summary statistics before and after cleaning, and (iii) graphical diagnostics illustrating how data screening mitigates mechanical outliers in the effective tax rate measure.

A1 Table. Sample Attrition by Cleaning Step

| Step | Cleaning criterion | Observations dropped | % of initial sample | Remaining observations |
| --- | --- | --- | --- | --- |
| 0 | Initial sample (non-financial A-share listed firms, 2019–2023) | – | – | 20,216 |
| 1 | Exclude firms with non-normal listing status (ST and *ST) | 748 | 3.70% | 19,468 |
| 2 | Exclude observations with negative depreciation of fixed assets | 20 | 0.10% | 19,448 |
| 3 | Exclude observations with effective tax rate greater than 1 | 319 | 1.58% | 19,129 |
| 4 | Exclude observations with anomalous “actual tax burden” (< 0 or > 1) | 47 | 0.23% | 19,082 |
| 5 | Exclude observations with negative pre-tax income | 3,430 | 16.97% | 15,652 |
| 6 | Final estimation sample | – | – | 15,652 |

Notes: This table documents the sequential screening procedure used to construct the estimation sample, following standard practices in the empirical tax and accounting literature.

Firms with non-normal listing status include those designated as ST or *ST by Chinese stock exchanges due to abnormal financial conditions or delisting risk.

The effective tax rate is defined as income tax expense divided by total profit.

The “actual tax burden” is measured as income tax payable divided by operating revenue.

Observations are dropped sequentially; percentages are calculated relative to the initial sample size of 20,216 observations.

Continuous variables are winsorized at the 1st and 99th percentiles; winsorization does not affect the number of observations.

A2 Table. Summary Statistics: Full Sample vs. Cleaned Sample

| Variable | Full sample: Mean | Full sample: SD | Cleaned sample: Mean | Cleaned sample: SD |
| --- | --- | --- | --- | --- |
| etr | 0.243 | 11.439 | 0.161 | 0.106 |
| digital | 0.289 | 0.453 | 0.278 | 0.448 |
| size | 22.265 | 1.348 | 22.366 | 1.344 |
| lev | 0.436 | 1.300 | 0.386 | 0.189 |
| cash | 0.167 | 0.133 | 0.180 | 0.132 |
| lnfad | 17.914 | 1.693 | 18.014 | 1.658 |
| capital | 0.191 | 0.148 | 0.190 | 0.144 |
| intang | 0.046 | 0.064 | 0.045 | 0.064 |
| ziji | 0.616 | 0.175 | 0.620 | 0.171 |
| chanye | 1.785 | 1.167 | 1.758 | 1.138 |
| lngdp | 29.351 | 0.687 | 29.375 | 0.657 |
| Observations | 20,216 |  | 15,652 |  |

Notes: This table compares summary statistics between the full sample and the cleaned estimation sample. The full sample includes all non-financial A-share listed firms prior to data cleaning, while the cleaned sample applies the exclusion criteria described in Appendix A. The large dispersion of the effective tax rate in the full sample reflects the presence of economically implausible values prior to data cleaning.


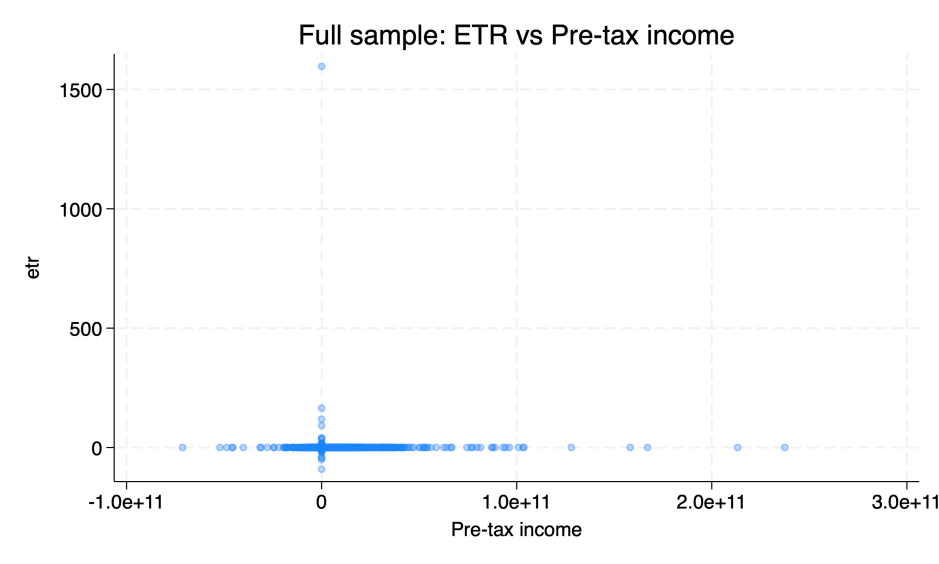


A1 Figure. Effective Tax Rate and Pre-tax Income: Full Sample


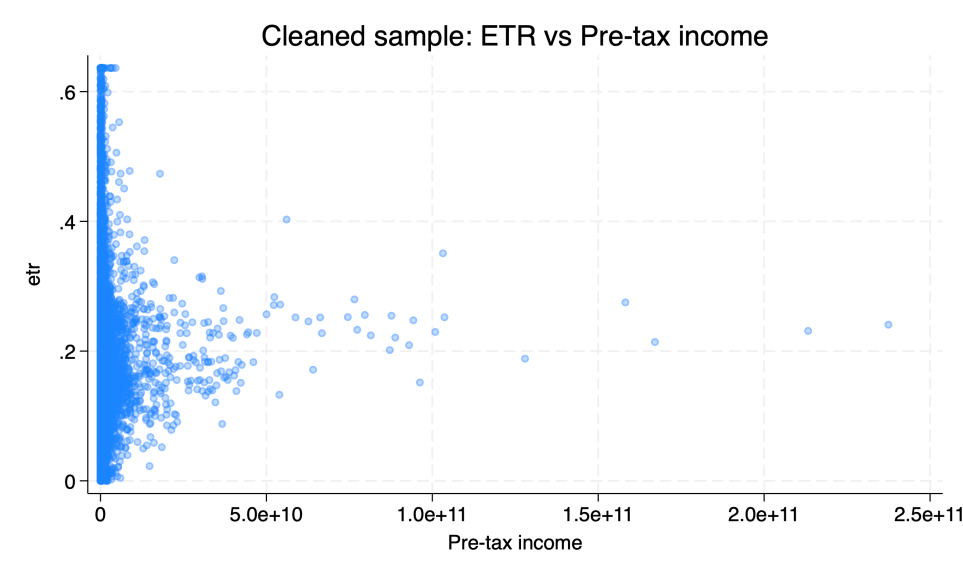


A2 Figure. Effective Tax Rate and Pre-tax Income: Cleaned Sample

**Appendix.B Addressing Potential Confounders**

During the sample period (2019–2022), China’s corporate income tax system remained institutionally stable, with no nationwide changes to statutory tax rates, tax base definitions, or core deduction rules, making it unlikely that observed within-firm changes in effective tax rates (ETR) are mechanically driven by contemporaneous tax policy reforms.

While the statutory corporate income tax system was stable, firms may nevertheless have been exposed to industry-specific and time-varying policy interventions or enforcement actions—such as sector-targeted inspections, compliance campaigns, or changes in regulatory intensity—that could coincide with the rollout of e-invoicing and confound the estimated effects.

To address this concern, we augment the baseline specification by introducing industry-by-year fixed effects, in addition to firm fixed effects. This specification absorbs all unobserved shocks that are common to firms operating within the same industry in a given year, thereby controlling for synchronized industry-level policy changes that might otherwise be correlated with the e-invoicing reform.

Under this more stringent control structure, the estimated effect of the e-invoicing reform on effective tax rates remains positive and statistically significant. As reported in Table B1, the coefficient on the reform indicator is 0.0072 and significant at the 1% level, closely aligned with the baseline estimates. This robustness indicates that the main results are not driven by co-occurring industry-level policy changes or enforcement shocks, but instead reflect firms’ behavioral responses to enhanced tax enforcement under e-invoicing.

B1 Table. E-invoicing reform and effective tax rates with industry-by-year fixed effects

| Variable | etr |
| --- | --- |
| treat | 0.00717***  (0.00227) |
| size | −0.00574  (0.00375) |
| lev | 0.0499***  (0.0176) |
| cash | −0.000655  (0.0146) |
| lnfad | 0.00595*  (0.00344) |
| capital | 0.0153  (0.0199) |
| intang | 0.0617  (0.0397) |
| ziji | 0.000833  (0.0550) |
| chanye | 0.00260  (0.00306) |
| lngdp | 0.0238  (0.0206) |
| Constant | −0.548  (0.580) |
| Industry × Year FE | yes |
| Firm FE | yes |
| Observations | 14,827 |
| Adj. R2 | 0.5703 |

Note: * p<0.1, ** p<0.05, *** p<0.01. Standard errors in brackets.

**Appendix.C Baseline and Robustness Results for Non-Regulated Industries**

Table C1. Summary Statistics: Non-Regulated Industries

| VarName | Obs | Mean | SD | Min | Max |
| --- | --- | --- | --- | --- | --- |
| etr | 11928 | 0.149 | 0.098 | 0.000 | 0.636 |
| digital | 11935 | 0.282 | 0.450 | 0.000 | 1.000 |
| size | 11935 | 22.294 | 1.328 | 19.973 | 26.401 |
| lev | 11935 | 0.378 | 0.180 | 0.056 | 0.836 |
| cash | 11935 | 0.182 | 0.134 | 0.014 | 0.635 |
| lnfad | 11935 | 17.998 | 1.707 | 14.279 | 22.545 |
| capital | 11935 | 0.196 | 0.146 | 0.002 | 0.654 |
| intang | 11895 | 0.047 | 0.070 | 0.000 | 0.936 |
| ziji | 11935 | 0.625 | 0.166 | 0.211 | 0.922 |
| chanye | 11935 | 1.755 | 1.149 | 0.900 | 5.690 |
| lngdp | 11935 | 29.407 | 0.642 | 26.952 | 30.239 |

Notes: This table reports summary statistics for firms in non-regulated industries.

Table C2. Baseline and robust results for non-regulated industries

| Variable | (1) etr | (2) etr | (3) etr2 | (4) taxdiff | (5) etr |
| --- | --- | --- | --- | --- | --- |
| digital | 0.0104**  (0.00443) | 0.0108**  (0.00418) | 0.00206*  (0.00104) | -0.0106**  (0.00424) | 0.0108***  (0.00383) |
| size |  | -0.00273  (0.00332) | 0.00562***  (0.00155) | 0.00833*  (0.00422) | -0.00273  (0.00527) |
| lev |  | 0.0421*  (0.0232) | -0.0257***  (0.00555) | -0.0473*  (0.0231) | 0.0421***  (0.0142) |
| cash |  | 0.00441  (0.0153) | 0.00783**  (0.00314) | -0.00370  (0.0147) | 0.00441  (0.00935) |
| lnfad |  | 0.00535**  (0.00196) | -0.00268***  (0.000724) | -0.00631***(0.00166) | 0.00535*  (0.00303) |
| capital |  | 0.0325  (0.0233) | -0.0160***  (0.00530) | -0.0228  (0.0243) | 0.0325*  (0.0191) |
| intangible~o |  | 0.0736*  (0.0419) | -0.0285*  (0.0159) | -0.0795*  (0.0452) | 0.0736**  (0.0341) |
| ziji |  | -0.00343  (0.0656) | 0.0185*  (0.00930) | -0.00730  (0.0637) | -0.00343  (0.0466) |
| chanye |  | -0.00124  (0.00401) | -0.00179**  (0.000728) | 0.000275  (0.00442) | -0.00124  (0.00601) |
| lngdp |  | 0.0484  (0.0349) | 0.00628  (0.00661) | -0.0715*  (0.0370) | 0.0484  (0.0317) |
| Constant | 0.147***  (0.00120) | -1.335  (1.000) | -0.238  (0.193) | 2.091*  (1.048) | -1.335  (0.930) |
| Year FE | yes | yes | yes | yes | yes |
| Firm FE | yes | yes | yes | yes | yes |
| N | 11,268 | 11,233 | 11,233 | 10,909 | 11,233 |
| Adj. R2 | 0.5210 | 0.5238 | 0.6586 | 0.4437 | 0.5238 |

Note: * p<0.1, ** p<0.05, *** p<0.01. Standard errors in brackets. Columns (1), (2), and (5) use etr as the dependent variable; Column (3) uses etr2 (income tax expense divided by operating revenue); Column (4) uses taxdiff (statutory minus effective tax rate). Standard errors are clustered at the province level in Column (2) and at the firm level in Column (5).


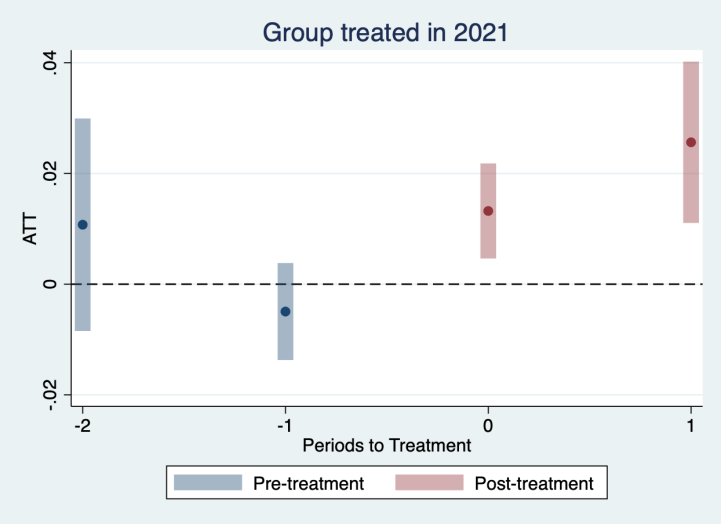


Figure C1. Event Study Estimates for Parallel Trends Validation for non-regulated industries
